# Impact of Pregnancy on Susceptibility and Severity of COVID-19: A Hospital-Based Prospective Observational Study

**DOI:** 10.7759/cureus.24281

**Published:** 2022-04-19

**Authors:** Kavita Khoiwal, Anoosha K Ravi, Shivaani Arora, Anmol Mittal, Amrita Gaurav, Latika Chawla, Rajlaxmi Mundhra, Anupama Bahadur, Prasan Kumar Panda, Jaya Chaturvedi

**Affiliations:** 1 Obstetrics and Gynaecology, All India Institute of Medical Sciences, Rishikesh, Rishikesh, IND; 2 Medicine, All India Institute of Medical Sciences, Rishikesh, Rishikesh, IND

**Keywords:** high-risk pregnancy, covid-19 india, disease severity of covid-19, covid-19 mortality, sars-cov-2 in pregnancy, infectivity potential of covid-19, infection in pregnancy

## Abstract

Objective: Pregnancy is a transient state of immunosuppression. The objective of this study was to ascertain whether pregnant women are more susceptible to coronavirus disease 2019 (COVID-19) than non-pregnant women and the impact of pregnancy on the severity of COVID-19 and associated morbidity and mortality.

Methods: A prospective observational study was performed at All India Institute of Medical Sciences (AIIMS) Rishikesh for a period of two months. A total of 42 and 33 COVID-19 positive women were included in the obstetric and non-obstetric cohorts respectively.

Results: Baseline characteristics were similar in both groups. Approximately 48% of the obstetric cohort had no COVID-19-related symptoms. Whereas, 100% of the non-obstetric cohort was symptomatic and had a significantly higher number of patients presenting with fever, cough, and breathlessness. The obstetric cohort had a significantly higher incidence of mild disease (p=0.009). In the obstetric cohort, the mean gestational age was 32.59 ± 2.57 weeks, with patients spread across all trimesters. Most of the patients with severe disease were in their second trimester. There was no difference in intensive care unit (ICU) admission, duration of ICU stay, duration of hospital stay, and mortality among both groups. A significantly smaller number of patients in the obstetric cohort required ventilatory support (p=0.0002). The maternal mortality rate was 16.67%. All of them had severe diseases requiring ICU admission. The cause of death was attributed to severe COVID pneumonia with septic shock in all cases. The mortality rate was comparatively higher (27.27%) in the non-obstetric group.

Conclusion: Pregnancy, unlike other immunocompromised conditions, does not seem to affect the prognosis of COVID-19 in terms of disease severity or mortality.

## Introduction

The WHO announced the coronavirus disease 2019 (COVID-19) pandemic on March 11, 2020. Since then, the world has seen its staggering effect, particularly in the healthcare system. Severe acute respiratory syndrome coronavirus 2 (SARS-CoV-2) and its various mutants have been responsible for more than one wave of infection globally. At the time of this study, India was in the midst of a second and a much larger wave and had already recorded a peak of nearly four lakh cases per day (WHO statistics).

Though, the cases of COVID-19 spread across all age groups and sex, higher susceptibility and poor outcomes have been observed in patients with immunocompromised status like cancer, old age, and diabetes [1]. Pregnancy is one such transient state of immunosuppression due to the physiological changes in the immune system [2-3]. The literature from the first wave across the globe has mixed opinions regarding the risks of COVID-19 in pregnancy and puerperium. There are large-scale studies wherein, the pregnant cohort does not behave much differently than the non-infected pregnant patients or age-matched non-pregnant patients with COVID-19 [4-6]. On the other hand, a higher risk for intensive care unit (ICU) admission, venous thromboembolism, and mortality were observed in infected pregnant women [7-11]. The literature on vertical transmission of SARS-CoV-2 is not conclusive, but scattered cases of such possibility cannot be ignored [12-14].

The second wave has already been dealt with in different parts of the world with a relatively more number of cases than the first wave. The susceptibility and associated morbidity and mortality have also been shown to be on the rise in the second wave in places like the United Kingdom and South Africa [15-16]. The reason for such a rise could be an overall increase in infection rate or a more virulent strain.

At the time of the present study, there has been no such study in India to evaluate the vulnerability of the pregnant cohort to this pandemic. Hence, this study was undertaken as an effort to ascertain whether pregnant women are more susceptible to COVID-19 than non-pregnant women and impact of pregnancy on the severity of COVID-19 and associated morbidity and mortality in India during the second wave.

## Materials and methods

A hospital-based prospective observational study was performed at a designated COVID-19 care center in the state of Uttarakhand in North India from the 1st of March 2021 to the 30th of April 2021. All obstetric patients with positive COVID-19 real-time reverse transcriptase-polymerase chain reaction (RT-PCR) reports were included irrespective of the period of gestation and pregnancy outcome. And, their data were compared with age-matched COVID-19 positive non-obstetric patients presented in the same duration. This study was conducted on hospitalized women of the reproductive age group (21-44 years). All pregnant women with COVID-19 positive status irrespective of the presence of symptoms and their severity were admitted. The indication for hospitalization in non-pregnant women was moderate to severe COVID-19 disease while asymptomatic and mild cases were managed at home only. Prior permission was obtained from the institutional ethical board for data collection and analysis (AIIMS/IEC/21/341).

Nasopharyngeal and oropharyngeal RT-PCR testing for COVID-19 was done for all patients at the time of admission as a screening procedure in the in-house ICMR (Indian Council of Medical Research) approved laboratory. The severity of the disease was categorized as mild, moderate, and severe based on clinical management protocol by the Ministry of Health and Family Welfare (MoHFW) version six. The management of both cohorts was done as per the institutional protocol conforming to the latest COVID-19 guidelines at the time.

Separate study personnel for each cohort collected the data in a predesigned proforma. The recruited patients were followed from admission till discharge or death while hospitalized. Data including demography, symptomatology, known co-morbidities, details of investigations and management, and duration of hospital stay were collected for both cohorts. For the obstetric cohort, additional data including the period of gestation, pregnancy-associated medical and surgical complications, and pregnancy outcomes were collected. Details of COVID-19 vaccination if any were collected for the non-obstetric cohort. Vaccination was not recommended for antenatal patients at the time of the study.

Patients underwent radiological evaluation with X-ray, ultrasonography, or CT chest whenever required. The choice of steroid and the dosage were guided by the latest guidelines at the time (Royal College of Obstetrics and Gynecology guidelines for obstetric cohort and MoHFW version six for non-obstetric cohort). The choice of antibiotics was based on local drug sensitivity trends. The ventilatory support if needed was given as per the clinical condition and weaned off as per recovery. All patients on steroids were monitored for blood glucose and insulin/oral hypoglycemic was added appropriately.

The primary objective of the study was to compare the disease severity and mortality due to COVID-19 in obstetric and non-obstetric cohorts. The secondary objective was to analyze the effect of pregnancy on symptomatology, presenting symptoms, ICU admission, ventilatory support, and hematological parameters of infection (neutrophils, lymphocytes, ferritin, CRP - C reactive protein, procalcitonin, and D-dimer), and duration of hospital stay. Study personnel who were not included in the data collection undertook the data analysis to avoid bias.

Statistical analysis

The continuous data were expressed as mean ± standard deviation (SD) or median (range) and compared by applying Student’s t-test and Mann-Whitney U test wherever applicable. The categorical data were expressed as a percentage and compared by Fisher’s exact test. p < 0.05 was considered significant.

## Results

A total of 42 and 33 COVID-19 positive women were included in the obstetric and non-obstetric cohorts respectively. Table 1 shows the baseline characteristics of the two groups. Both groups were comparable in age and pre-existing comorbidities. Among the two cancer patients in the non-obstetric cohort, one had metastatic carcinoma breast and the other had metastatic adenocarcinoma ovary. At the time of the study, only one patient from the non-obstetric cohort had received COVID vaccination. The median age of the obstetric cohort was 29 years (range 21-44) and the non-obstetric cohort was 32 years (range 21-38). About 26.19% and 12.12% of patients were referred from another center in the obstetric and non-obstetric cohort, respectively. It was noted that 47.62% of the obstetric cohort had no COVID-19-related symptoms. Whereas, 100% of the non-obstetric cohort was symptomatic. The non-obstetric cohort had a significantly higher number of patients presenting with fever, cough, and breathlessness. However, the recorded temperature on presentation was similar in both groups. Additionally, patients from the non-obstetric cohort had a significant respiratory compromise in terms of respiratory rate and oxygen saturation at the time of presentation. Patients from the obstetric cohort had a significantly higher incidence of mild disease (p = 0.009).

**Table 1:** Baseline characteristics of the study participants. Data expressed as mean±SD or n (%) SD, standard deviation; CKD, chronic kidney disease

Variables	Obstetric cohort (n=42)	Non-obstetric cohort (n=33)	p value
Age, years	30.24 ± 4.52	31.73 ± 4.39	0.15
Co-morbidities, n (%)			
Hypertension	1 (2.38)	2 (6.06)	0.57
Diabetes	3 (7.14)	2 (6.06)	1
Thyroid disorders	8 (19.05)	3 (9.09)	0.32
History of Tuberculosis	0	1 (3.03)	NA
Others	Hepatitis B: 1 (2.38)	Cancer: 2 (6.06), CKD: 1 (3.03), Hepatitis B: 1 (3.03)	-
Symptoms, n (%)			
No symptoms	20 (47.62)	0	NA
Fever	17 (40.48)	28 (84.85)	0.0001*
Cough	16 (38.09)	29 (87.88)	0*
Breathlessness	13 (30.95)	25 (75.76)	0.0002*
Diarrhea	4 (9.52)	6 (18.18)	0.31
Chest pain	0	2 (6.06)	NA
Sore throat	1 (2.38)	1 (3.03)	1
Altered sensorium	0	3 (9.09)	NA
Nausea/vomiting	2 (4.76)	1 (3.03)	1
Fatigue	11 (26.19)	0	NA
Duration of onset of symptoms to hospitalization (days)	3 (1-10)	5 (2-15)	0.06
Baseline clinical assessment at presentation			
Pulse rate (beats/min)			
<60	0	0	NA
60-100	33 (78.57)	19 (57.58)	0.07
>100	9 (21.43)	14 (42.42)	
Mean arterial pressure (mmHg)			
<70	1 (2.38)	1 (3.03)	1
70-100	38 (90.48)	26 (78.79)	0.19
>100	3 (7.14)	6 (18.18)	0.17
Respiratory rate (/min)			
<24	36 (85.71)	20 (60.6)	0.017*
>24	6 (14.29)	13 (39.39)	
Temperature (degree celsius)			
<38	38 (90.48)	32 (96.97)	0.37
>38	4 (9.52)	1 (3.03)	0.37
Spo2			
<90%	6 (14.29)	9 (27.27)	0.24
90%-93%	5 (11.90)	12 (36.36)	0.02*
>93%	31 (73.81)	12 (36.36)	0.002*
Disease severity			
Mild	25 (59.52)	9 (27.27)	0.009*
Moderate	6 (14.29)	9 (27.27)	0.24
Severe	11 (26.19)	15 (45.45)	0.09

In the obstetric cohort, the mean gestational age was 32.59 ± 2.57 weeks, with patients spread across all trimesters (two from first, nine from second, and the rest from the third trimester) as shown in Table 2. Among patients with severe COVID, 81.82% were in the second trimester and 18.18% were in the third trimester. Out of 42 antenatal patients, 25 had pregnancy-associated complications. By the time of study reporting, 29 patients had a termination of pregnancy (21 had a vaginal delivery, seven had a cesarean delivery, and one had dilatation and curettage) resulting in 26 live births with an average birth weight of 2594.81 ± 662.10 g. There were two early neonatal deaths, one was associated with antepartum hemorrhage and severe prematurity, and the other was associated with acute fatty liver of pregnancy. Both mothers had mild COVID disease. A total of seven patients had a continuation of pregnancy (four with severe, one with moderate, and two with mild disease). The decision to continue of pregnancy was made by the mother after understanding all possible risks and complications including the risk of vertical transmission.

**Table 2:** Maternal and perinatal characteristics of the obstetric cohort. Data expressed as mean ± SD or n% SD, standard deviation

Variables	Obstetric cohort (n=42)
Gestational age, weeks (mean±SD)	32.59 ± 2.57
First trimester (0-12 weeks 6 days)	2 (4.76%)
Second trimester (13 to 27 weeks 6 days)	9 (21.43%)
Third trimester (28 to 41 weeks)	31 (73.81%)
Pregnancy-associated illness	
Pre-eclampsia	3 (7.14%)
Eclampsia	2 (4.76%)
Gestational diabetes mellitus	4 (9.52%)
Hypothyroidism	8 (19.05%)
Anemia	7 (16.67%)
Acute fatty liver of pregnancy	1 (2.38%)
Pregnancy-associated complications	
Term or preterm rupture of membranes	6 (14.28%)
Fetal growth restriction	5 (11.90%)
Oligohydramnios	9 (21.43%)
Intrauterine death	5 (11.90%)
Outcome	
Continuation of pregnancy	7 (16.67%)
Term delivery	19 (45.23%)
Preterm delivery	9 (21.43%)
Dilatation and curettage	1 (2.38%)
Birth weight (g)	2594.81 ± 662.10

In the obstetric cohort, a subgroup analysis was performed to compare severe and non-severe diseases (Table 3). The age and duration of symptoms were similar in both subgroups. However, a significant difference was observed in gestational age (p = 0.00001) with most of the patients with the severe disease having lesser gestational age. Approximately 81.82% of patients with severe disease had pregnancy-associated complications. All patients with intrauterine fetal death (IUFD) had severe COVID disease. The incidence of oligohydramnios and preterm delivery was comparable. Whereas, all cases of fetal growth restriction and premature rupture of membranes had the non-severe disease.

**Table 3:** Comparison of obstetric patients with non-severe and severe diseases. Data expressed as mean ± SD, n (%), or median (range) SD, standard deviation; NA, not applicable

Parameter	Obstetric patients with non-severe disease (n=31)	Obstetric patients with severe disease (n=11)	p value
Age (years)	30.35 ± 4.79	29.91 ± 3.86	0.78
Duration of symptoms (days)	2.5 (1-10)	3 (1-8)	0.95
Gestational age	35.33 ± 2.78	24.89 ± 2.65	<0.00001*
Intrauterine death	0	5 (45.45)	NA
Fetal growth restriction	5 (16.12)	0	NA
Premature rupture of membranes	6 (19.35)	0	NA
Oligohydramnios	6 (19.35)	3 (27.27)	0.67
Preterm delivery	8 (25.81)	1 (9.09)	0.40

Table 4 shows the comparison of the course of disease in the obstetrics and non-obstetric cohorts. There was no difference in ICU admission, duration of ICU stay, duration of hospital stay, and mortality among both groups. A significantly smaller number of patients in the obstetric cohort required ventilatory support (p = 0.0002). However, there was a higher incidence of neutrophilia, raised CRP, and raised D-dimer in the obstetric cohort. Lymphopenia was present in about 73.81% of the obstetric cohort and 57.58% of the non-obstetric cohort, the difference was not significant. Mean serum creatinine was significantly higher in the non-obstetric cohort. When one patient from the non-obstetric cohort with chronic kidney disease (CKD) was excluded, the difference between the two groups with serum creatinine was not significant (p = 0.06). Additionally, serum ferritin levels were higher in the non-obstetric cohort. It was observed that the usage of steroids in the non-obstetric cohort was more liberal than in the obstetric cohort. All 33 patients in the non-obstetric cohort received antibiotics, whereas only 38.09% of patients in the obstetric cohort received antibiotics. The choice of antibiotics was based on local drug sensitivity and included macrolides, fluoroquinolones, and a combination of penicillin and beta-lactamase inhibitors. The choice of antivirals in three patients in the non-obstetric cohort was Remdesivir with or without Tocilizumab.

**Table 4:** Comparison of the course of disease in the obstetric and non-obstetric cohorts. Data expressed as mean ± SD, n (%), or median (range) SD, standard deviation; NRBM, non-rebreather mask; NIV, non-invasive ventilation; SGOT, serum glutamic oxaloacetic transaminase or aspartate transaminase; SGPT, serum glutamic pyruvic transaminase or alanine aminotransferase

Characteristic	Obstetric cohort (n=42)	Non-obstetric cohort (n=33)	p value
ICU admission	11 (26.19)	12 (36.36)	0.45
Duration of ICU stay, days	6 (1-11)	5 (1-19)	0.81
Ventilatory support			
None	30 (71.43)	9 (27.27)	0.0002*
Face mask/nasal prongs	3 (7.14)	7 (21.21)	0.09
NRBM	0	7 (21.21)	NA
NIV	2 (4.76)	2 (6.06)	1
Invasive ventilation	7 (16.67)	8 (24.25)	0.56
Investigations			
Hemoglobin (g/dL)	10.99±1.49	11.28±1.49	0.41
Total leucocyte count (/cumm)	10677.90±3799.64	10661.56±4985.45	0.98
<4000	0	1 (3.03)	NA
4000-11000	27 (64.29)	18 (54.55)	0.47
>11000	15 (35.71)	14 (42.42)	0.63
Neutrophils (%)			
<40%	0	1 (3.03)	NA
40-70%	3 (7.14)	9 (27.27)	0.02*
>70%	39 (92.86)	23 (69.70)	0.01*
Lymphocytes (%)			
<20%	31 (73.81)	19 (57.58)	0.14
20%-40%	11 (26.19)	13 (39.39)	0.31
>40%	0	1 (3.03)	NA
Platelet (/cumm)			
<1.5 lakh	13 (30.95)	8 (24.24)	0.60
>1.5 lakh	29 (69.05)	25 (75.76)	
Serum creatinine (mg/dL)	0.76±0.18 (0.34-1.13)	1.42±1.96 (0.5-9.59)	0.03*
SGOT (U/L)			
<40	20 (47.62)	18 (54.55)	0.64
>40	22 (52.38)	15 (45.45)	
SGPT (U/L)			
<40	26 (61.91)	18 (54.55)	0.63
>40	16 (38.09)	15 (45.55)	
Ferritin (ug/dL)	236.78±249.29	584.92±391.47	0.01*
Procalcitonin (ng/mL)			
<0.5	38 (90.48)	31 (93.94)	0.68
>0.5	4 (9.52)	2 (6.06)	
CRP (mg/dL) >0.6	15 (35.71)	2 (6.06)	0.002*
D-dimer (mg/dL)	2.54±3.79	1.19±0.89	0.02*
<0.5	14 (33.33)	14 (42.42)	0.47
0.5-2	18 (42.86)	17 (51.52)	0.49
2-4	7(16.67)	2 (6.06)	0.28
>4	3 (7.14)	0	NA
Abnormal radiology (chest X-ray or CT scan)	13 (30.95)	17 (51.52)	0.09
Treatment			
Steroid	17 (40.48)	26 (78.79)	0.001*
Clexane	34 (80.95)	22 (66.67)	0.18
Antivirals	0	3 (9.09)	NA
Antibiotics	16 (38.09)	33 (100)	<0.00001*
Single	8/16 (50)	8/33 (24.24)	0.10
Combination	8/16 (50)	25/33(75.76)	
Vasopressor	7 (16.67)	4 (12.12)	0.74
Duration of hospital stay	6 (2-12)	6 (1-32)	0.08
Mortality	7 (16.67)	9 (27.27)	0.39

The maternal mortality rate in the present study was 16.67%. All of them had severe diseases requiring ICU admission. Lymphopenia was present in all cases. Out of them, five had IUFD. The cause of death was attributed to severe COVID pneumonia with septic shock in all cases. The mortality rate was comparatively higher (27.27%) in the non-obstetric group, however, the difference was not statistically significant.

## Discussion

The present study was the first of its kind in India comparing obstetric and non-obstetric cohorts with COVID-19. It was observed that pregnant women mostly presented with mild COVID disease and there was no difference in mortality when compared with age-matched infected non-pregnant patients. In this study, the incidence of symptoms like fever, cough, and breathlessness was more prevalent in non-obstetric patients and they commonly presented with tachypnea and lower oxygen saturation. This was reflected in the relatively frequent requirement of ventilatory support in non-obstetric patients. However, there was no difference in ICU admission or duration of ICU stay in both groups. The patients from the obstetric cohort had higher neutrophilia, CRP, D-dimer, and lower ferritin levels compared to the non-obstetric cohort. Both groups had comparable lymphocyte and procalcitonin. There was no significant difference in hospital stay between the two groups. It was also observed that pregnant women with severe disease mostly were in the second trimester and they had a higher incidence of IUFD.

Almost half of the patients from the obstetric cohort were asymptomatic, unlike the non-obstetric cohort where all of them had symptoms. This might be because, asymptomatic non-pregnant patients were managed by home isolation and also, asymptomatic pregnant women got admitted for pregnancy-associated complications or delivery. The finding of a more asymptomatic course in pregnancy was supported by a systematic review by Jafari et al. [17]. Vizheh et al. reported a significantly higher prevalence of cough and dyspnea in non-pregnant women in a retrospective cohort study [18], similar to our study.

The present study included pregnant patients from all trimesters. Though the majority had only mild disease, all nine patients in the second trimester had severe disease (p = 0.00001). All pregnant patients with severe disease were symptomatic and more than 80% had pregnancy-associated complications. Additionally, all cases of IUFD and maternal mortality were associated with severe disease. This observation seems unique to this study as the majority of previous studies had included pregnant women mostly from the third trimester [5]. In a study by Qiancheng et al. [19], all women in the first and second trimesters had a termination of pregnancy due to concerns of COVID-19.

There was a higher neutrophilia (p = 0.01), D-dimer (p = 0.02), lymphopenia (p = 0.14), and raised CRP (p = 0.002) in obstetric cohort. This finding was consistent with other studies [17-19]. Elevated CRP observed in most studies could be because of pregnancy itself [20]. Higher serum creatinine in the non-obstetric cohort could be the influence of a case of chronic kidney disease as once this patient was excluded, the difference between the two groups was not significant. 

In the present study, the requirement of ICU care was similar in both groups while ventilatory support was less required in obstetric patients. This observation was correlating with the finding of increased respiratory compromise in non-obstetric patients. Such a comparable need for ICU was also reported by Vizheh et al. [18]. On the other hand, Ellington et al. reported 1.5 times higher ICU admission in pregnant COVID-19 positive patients with no difference in mortality [9]. Similarly, an odds ratio of 2.13 was reported for ICU admission in pregnant women compared to non-pregnant women in a systematic review and meta-analysis [11]. This finding could be owed to a higher incidence of obesity, diabetes, hypertension, and older age in pregnant women in their study [11].

Lesser usage of steroids and antibiotics in pregnant patients in the present study could be explained by a higher incidence of mild disease in the obstetric cohort. Another reason for such difference could be the lack of standard guidelines or ever-changing guidelines in the management of COVID in pregnancy.

There was no difference in mortality between the two groups similar to previous studies [9, 18-19]. Whereas, Villar et al. reported that the pregnant women with COVID-19 were 22 times more likely to die (1.6% mortality) than pregnant women without COVID-19 in a multinational cohort study (INTERCOVID) [7]. The higher mortality rate could have been influenced by poor ICU care in less developed regions included in this study and the higher prevalence of pre-existing medical conditions (obesity, diabetes, hypertension, and chronic respiratory conditions) in pregnant women with COVID-19.

The maternal mortality with COVID-19 in the present study was 16.67% which was much higher than that observed by previous studies [7, 17-18]. This could be explained by the higher load of cases in the second wave of infection in India. And also, as the study institution was a tertiary center, it accepted all referred complicated cases.

Thus, the present study suggests that pregnancy does not increase the infective potential of SARS-CoV-2 and the severity of COVID-19. This result is consistent with studies from various parts of the world mostly conducted during the first wave of infection [4-6]. Whereas, we performed this study in the midst of second wave. One alarming finding from the obstetric cohort in the present study was that severe disease seems to be concentrated in the mid-trimester resulting in higher complications like IUFD and maternal mortality.

The strengths of the study included its prospective nature and recruitment of obstetric patients from all trimesters. The study was set in a single center thus there was consistency in the management of patients.

The findings of this study are subject to the following limitations. Being a tertiary care hospital, all referred cases (severe and complicated cases) were accepted. While asymptomatic or mild cases in the non-obstetric cohort were managed by home isolation. Hence, our study population represented more severe cases, which might have led to a higher mortality rate compared to other similar studies. Additionally, there is a possibility of selection bias as all COVID-19 positive obstetric women were admitted irrespective of symptoms whereas only moderate and severe cases were admitted in the non-obstetric cohort. Virus clearance time was not studied as COVID testing was not recommended before discharge. Additionally, the data presented in the study only included until the discharge of the patient. Follow-up data for complications or new symptoms associated with illness or its treatment like mucormycosis was not part of the observation. COVID-19 being a novel illness, the treatment strategies are constantly updated and studies involving pregnant COVID positive patients for treatment are limited. More focused studies to develop management protocols in pregnant patients are required. In addition, a higher rate of complications (IUFD) and maternal mortality in mid-trimester need to be evaluated further with targeted studies on pregnant women in mid-trimester.

## Conclusions

Pregnancy, unlike other immunocompromised conditions, does not seem to affect the prognosis of coronavirus infection in terms of disease severity or mortality. The majority of these cases have milder disease and more favorable presenting features. This could be owed to more frequent testings and routine admissions in cases of pregnant women with COVID illness. The infection in the second trimester seems to be associated with higher complications like intrauterine death and maternal mortality. This finding needs to be evaluated further with more focused studies on pregnant women in mid trimester. This like previous studies continues to be an advisory for women planning a pregnancy or currently pregnant to take adequate strict measures for prevention of infection. Also, more studies are required for management protocols and vaccination effects in pregnant women with COVID-19.
